# Molecular Dynamics Simulation and Experimental Study of Mechanical Properties of Graphene–Cement Composites

**DOI:** 10.3390/ma17020410

**Published:** 2024-01-13

**Authors:** Henggan Li, Fupeng Lan, Yulin Wang, Xiaotian Lin, Yan Zhao, Qi Zhen, Dehong Chen

**Affiliations:** 1 Department of Civil Engineering and Architecture, Wuyi University, Nanping 354300, China; lhg@wuyiu.edu.cn (H.L.); ylwanghm@163.com (Y.W.); 18950630981@163.com (Q.Z.); 15759803268@163.com (D.C.); 2 Engineering Research Center of Prevention and Control of Geological Disasters in Northern Fujian, Fujian Province Higher Education Institutes, Nanping 354300, China; 3 Department of Engineering, Nanping Wuyi Development Group Co., Ltd., Nanping 353000, China; fjshlfp@163.com

**Keywords:** molecular simulation, calcium silicate hydrate, graphene, mechanical properties

## Abstract

To investigate the mechanical properties of graphene (G) and calcium silicate hydrate (C-S-H) composites in different directions, molecular dynamics (MD) simulations and experiments were used, and the effects of temperature, loading rate, and graphene defects were also investigated. The experimental results show that the addition of graphene can improve the flexural, compressive, and tensile strength of the composite. The results of molecular dynamics simulation show that the addition of graphene in x and z directions can enhance the tensile strength of G/C-S-H in three directions, while the addition of graphene in y direction can reduce the tensile strength of G/C-S-H. At the same time, the tensile strength of G/C-S-H decreases with the increase in temperature and increases with the increase in loading rate. Meanwhile, the mechanical properties of G/C-S-H can be improved using a certain concentration of monatomic vacancy defects, diatomic vacancy defects, and S-W defects.

## 1. Introduction

C-S-H gel, which makes up more than 60% of cement composite materials, is one of the key elements of cement hydration materials and the primary factor in cement’s mechanical properties. Researchers have recently developed a number of microscopic models to analyze the mechanical characteristics of C-S-H in addition to macro-scope studies that study the mechanical properties of cement. Many mineral analogs of CSH gels, including tobermorite and metajennite, were proposed by Richardson et al. in 2007 [1]. Tobermorites are hydrating silicates that exist naturally and the main model used in molecular dynamics simulations of cement. They are remarkably comparable to the C-S-H generated by cement hydration. They can be classified into three categories [2], namely tobermorite 9, 11, and 14, depending on the varying basement spacing that corresponds to the various levels of hydration. Among them, tobermorite 11 Å is a simulation model widely used at present, which has two structures: the Hamid structure [3] and the Merlino structure [4]. The intermediate layers of the Merlino structure include Si-O-Si connections, while the Hamid structure is thought to consist of a single layer of calcium silicate. As molecular simulation techniques develop, an increasing number of academics are putting out increasingly ideal C-S-H models. Abdolhosseini Qomi and Hou et al. [5,6,7,8], for instance, enhanced the conventional modeling technique and produced new C-S-H models, which improved the consistency between the macroscopic experimental data and the microscopic behavior of C-S-H. Currently, most researchers consider C-S-H gels to be a structure intermediate between ordered crystals and disordered glasses, which are characterized by a random distribution of calcium atoms, a layered structure, and incompletely amorphous silicate chains [9].

The study of the microscopic properties of C-S-H involves mechanics, electricity, heat, and other aspects, among which the study of mechanical properties has attracted the interest of a large number of scholars. Researchers have used MD simulations to study the C-S-H structure and obtained results on mechanical properties that could not be obtained from macroscopic experiments, and these results allow for a better explanation of the macroscopic properties. In terms of mechanical properties, the compression property of C-S-H is found to be stronger than its tensile property. As a result, there has been more extensive research on the compression property, while the tensile property has received comparatively less attention. Great efforts have been made to improve the tensile properties of cement through a variety of techniques. Researchers have improved the mechanical properties of concrete by adding various types of materials, for example the addition of an aluminum phase [10], nanofibers and carbon nanotubes [11], graphene and graphene oxide [12], and epoxy and polyacrylics [13] can improve the mechanical properties of C–S–H. A molecular dynamics analysis of the structure of tobermorite 11A with a Ca/Si ratio of 1.6 to was performed by Hou et al. [6], and they obtained its tensile mechanical properties in different orientations and also investigated the effect of strain rate on the uniaxial tensile properties. Murray et al. investigated the mechanical properties, such as compressive and tensile strength, of crystalline and dimerized silicate chain C-S-H models under uniaxial tension [14]. Bauchy et al. [15] carried out MD simulations of defect-containing tobermorillonite 11 Å and obtained the fracture properties of C-S-H. The fracture properties of the tobermorillonite 11 Å model are shown in the following table. The study used the ReaxFF force field and obtained some results that could not be obtained from macroscopic experiments.

In recent years, the properties of cement-based composites have attracted the attention of many researchers. The experimental results of many researchers have shown that G/GO can enhance the mechanical properties of cementitious materials. By adding graphene oxide with a content of 0.05% to the cement material, Zhu Pan et al. [16] found that the mechanical properties of the composite were significantly enhanced. The results of Li et al. [17] showed that the addition of 0.04% graphene oxide to cement paste can increase its compressive stress by 14%. Yu et al. [18] found that the yield stress of cement composites can be increased by more than four times with the addition of 0.08% graphene oxide. Similarly, by adding graphene oxide with a content of 0.04% to cement, Sabziparvar et al. [19] found that the compressive stress of composite materials with different curing times was greatly improved. In a study by Hou et al. [20], it was found that the flexural stress of the material increased by 11.62% after incorporating 0.16 wt% of GO in the cement. The researchers concluded that the incorporation of G/GO has a facilitating effect on the hydration reaction and can reduce the defects of the composites, thus enhancing the mechanical properties of the cement materials.

However, macroscopic experiments cannot obtain molecular-scale information, so MD simulations are needed to observe the interactions between cement microstructures and provide microscopic guidance for enhancing the mechanical properties of cement materials. In recent years, MD simulation of G/GO-C-S-H composites has received extensive attention. Graphene is a single-layer hexagonal honeycomb lattice structure formed by sp^2^ hybrid bonding of carbon atoms. Graphene has excellent mechanical properties and is widely used in various engineering structures [21]. The interface characteristics of G/GO/C-S-H have been studied by Yi Yang and Yang Zhou [22,23]. MD simulations were run to learn more about the interactions between G/GO and C-S-H at the molecular level. Alkhateb et al. [24] investigated the interfacial properties between graphene and C-S-H structures using MD simulations, and the results showed that graphene improves the mechanical properties of composites by improving the G/C-S-H interfacial properties. Mao et al. [25] showed that the type of defects in graphene and the content of functional groups in graphene oxide can significantly impact the mechanical properties of composites.

So far, although some studies have been conducted on the tensile behavior of C-S-H gels at the molecular level, fewer of them have comparatively studied the tensile behavior of G/C-S-H composites in the three directions, and the effects of temperature, loading rate, and defects of graphene on the tensile behavior have not been considered comprehensively, which affects the overall understanding of the mechanical properties of C-S-H and graphene composites.

In this study, an experiment and MD simulations were used to investigate the mechanical properties of G/C-S-H with the introduction of graphene in the x, y, and z directions. The C-S-H structure was first modeled based on Pellenq et al. [26]. Subsequently, the established graphene model was embedded in the C-S-H and used to simulate the uniaxial tensile properties of G/C-S-H composites in the x, y, and z directions. The stress–strain curves during the tensile process were obtained. The effect of temperature, loading rate, and defects in graphene, including monovacancy defects, divacancy defects, and S-W defects, on the mechanical properties of G/C-S-H were also studied.

## 2. Experimental and Simulation Methods

### 2.1. Experimental Methods

The mechanical properties of C-S-H and G/C-S-H were measured with a hydraulic universal testing machine. The graphene used in the experiment is a single-layer graphene produced by Nanjing Xianfeng Nanomaterials Technology Co., Ltd. (Nanjing, China) The size of the graphene sheet is 0.5~5μm, and the thickness is 0.8–1.2 nm. In order to create the dispersant solution for the test, 100 mL of pure water and the weighted dispersant polyethylpyrrolidone K30 (PVP-K30) were first poured into a container. The weighed graphene was added into the dispersant solution, and in order to create the dispersant solution for the test, 100 mL of pure water and the weighted dispersant PVP-K30 were first poured into a container. In order to fully immerse the weighted graphene in the dispersant solution, the mixture was first artificially agitated before being placed inside the ultrasonic device for ultrasonic dispersion via a 30 min ultrasonic period. The graphene was removed following the ultrasonic treatment and left until use for 10 min.

Then, the weighed cement was added to the mixing pot and stirred at a slow speed. Meanwhile, the dispersed graphene mixed solution was slowly added, and then the remaining pure water was added and the mixture was stirred rapidly for 90 s. The mixed cement slurry was poured into the mold and vibrated for 60 s. The mold was removed one day later and maintained for 28 days.

For the compressive and flexural strength test, the size of the sample was 40 mm × 40 mm × 160 mm, and the loading speed was 2400 kN/s. For the tensile strength test, the sample was used as a “bone” mold, the size of both ends was 50 mm × 100 mm × 50 mm, the size of the middle was 250 mm × 50 mm × 50 mm, and the loading speed was 1500 kN/s. In order to avoid accidental error in the experiment, three groups of samples were used for the experiment and their average values were taken as the results.

### 2.2. Simulation Methods

**Figure 1:**
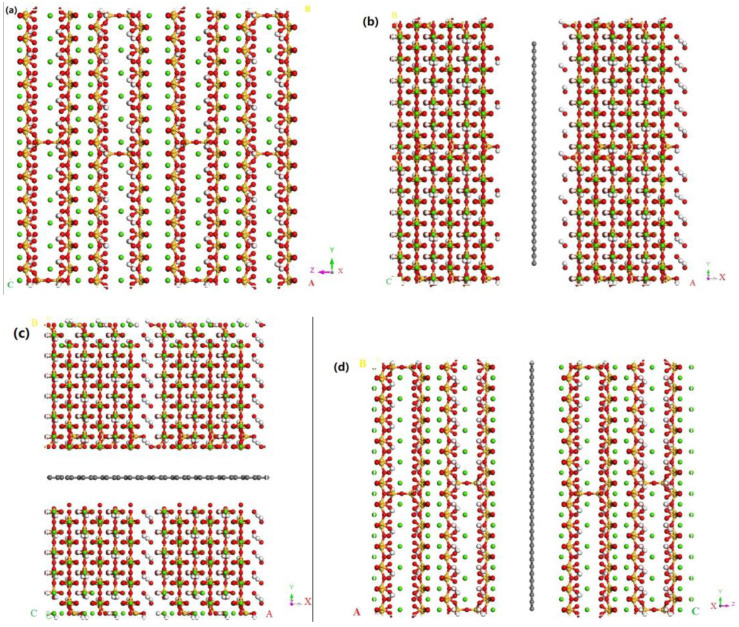
(**a**–**d**). Models of C-S-H and G /C-S-H composites: (**a**) C-S-H. (**b**–**d**) show the G/C-S-H with graphene placed in the middle of the x, y, and z directions of the C-S-H model, respec tively. The green balls represent the calcium atoms (Ca), the yellow balls represent the silicon atoms (Si), the red balls represent the oxygen atoms (O), and the white balls represent the hydrogen atoms (H). Thelocation relation of the letter A, B and C in the figure is the same as the location relation of the *x*-, *y*- and *z*-axis.

In this study, the C-S-H model was constructed based on the modeling idea of Pellenq [26] as follows: First, a 4a × 3b × 1c supercell structure of the monoclinic 11 Å tobermorite crystal model [3] was established as the initial structure, in which the water molecules and the hydrogen atoms in the OH group were deleted. In order to satisfy the Qn distribution and C/S ratio, a part of SiO_2_ and Si_2_O_5_ in the model was randomly deleted from the silicon chain. After that, C-S-H was allowed to fully absorb water molecules via the GCMC method [27], and then the structure was fully equilibrated under NPT system synthesis. The C-S-H model was thus constructed. The chemical composition of the C-S-H model is (CaO) 1.67 (SiO_2_) (H_2_O) 1.69, density 2.45 g/cm^3^. Qn distribution is Q0 = 11.6%; Q1 = 65.1%; Q2 = 23.3%. These characteristics are generally similar to the experimental data. The initial tobermorite model was expanded periodically along the x, y, and z directions to obtain a C-S-H model of size 40 Å × 44 Å × 45 Å. And finally, the graphene model was added to the C-S-H model. First, the graphene model was built based on a single-layer graphene sheet with a lattice constant of 3.4 Å × 2.46 Å × 2.46 Å, which was repeatedly enlarged in the x, y, and z directions to produce the final graphene structure of size 3.4 Å × 39 Å × 44 Å. Then, a 10 Å wide crack was cut along the middle of the three directions of the model, and the monolayer graphene sheet was embedded in this nanofracture. The model is shown in Figure 1.

The C-S-H simulation process was carried out under the ClayFF force field [28], which is an ionic force field whose force field parameters can be obtained through calculations of density generalization theory and quantum chemistry. Under this force field, all atoms are able to translate completely freely and the system can reach equilibrium quickly because all atoms are represented by point charges and there are interactions between point charges. The graphene interatomic interactions are described by the AIREBO potential function. Periodic boundary conditions were used for the whole system, which could effectively eliminate the boundary effects of the simulated system.

In this paper, the numerical simulation of mechanical properties of C-S-H and G/C-S-H molecular structures was carried out using the MD method. The simulation software used was LAMMPS-2017. The time step of the simulation was set to 0.0005 ps, and the temperature and intensity during the simulation were constant (NPT).

The simulations were performed as follows: First, the model was optimized for energy minimization prior to the simulation of the uniaxial tensile properties. Subsequently, the model was relaxed through NPT synthesis, and during the relaxation the model was warmed up and cooled down. The model was warmed up to 800 K from the initial simulation temperature and then cooled down to the previous simulation temperature after a period of relaxation to make it fully relaxed, while the pressure in the x, y, and z directions was controlled to zero. Finally, the C-S-H and G/C-S-H composites were loaded with tension by applying a strain rate load in one direction, respectively. The pressure in the other direction was always controlled to zero, while the pressure in the tensile direction was not controlled.

## 3. Result and Discussion

### 3.1. The Mechanical Behavior in Experiment

**Figure 2:**
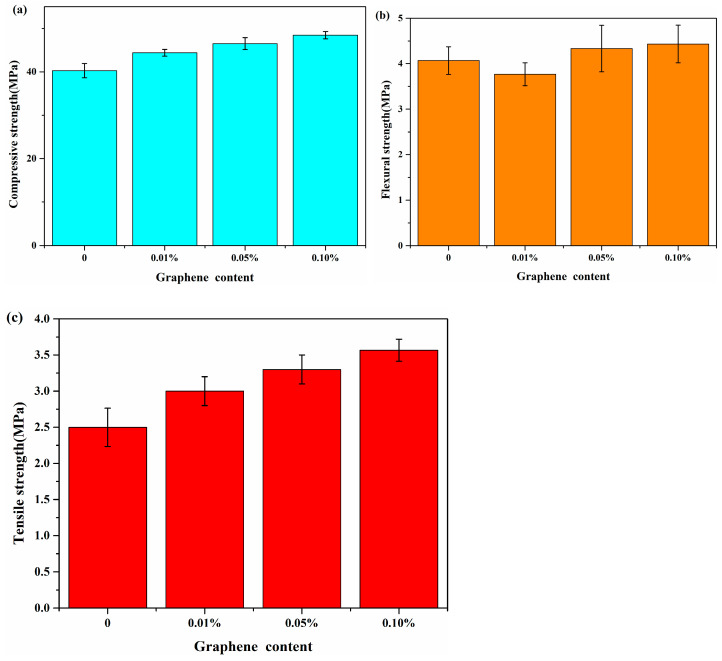
(**a**–**c**) Compressive, flexural, and tensile strength of graphene and cement composite varies with the content of graphene.

**Figure 3:**
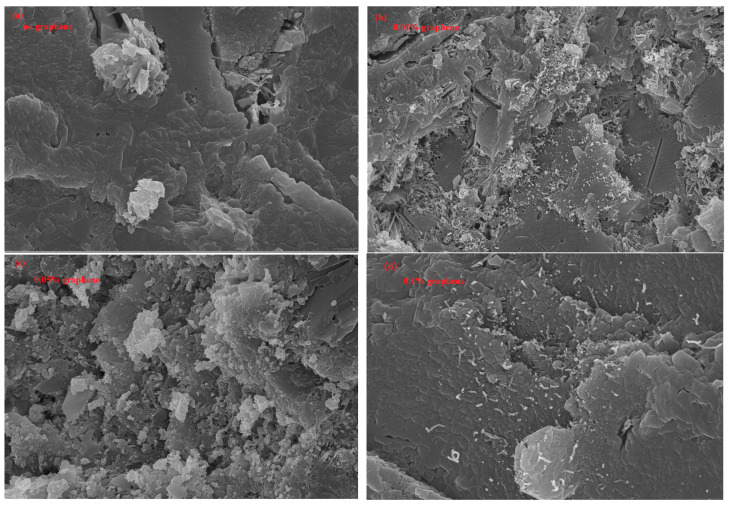
(**a***–***d**) Electron microscopic images of cement paste with graphene.

The compressive, flexural, and tensile stress test results of G/C-S-H at 28 days are shown in Figure 2. As can be seen from the figure, the compressive stress, flexural stress, and tensile stress gradually increased with the increase in graphene content, and the highest compressive strength is 48.9 MPa, which is 21.9% higher than that of C-S-H. The highest flexural strength is 4.6 MPa, which is increased by 12.19% compared with C-S-H. The maximum tensile strength is 3.5 MPa, 34.6% higher than that of C-S-H. This is also consistent with the results of the mechanical properties of G/GO/C-S-H at 14 days obtained by Hou et al. [20]. The reason for the enhancement of the mechanical properties of G/C-S-H is that the excellent mechanical properties of graphene have an effect on the performance of composite materials. On the other hand, it can be found from the electron microscope images that graphene has a bridging effect on the control of microcracks in the cement matrix (Figure 3). Figure 3a–d are the SEM images of the samples in the experiment. It can be found from the figures that with the increase in graphene content, the microstructure of the cement paste gradually becomes more dense. More obvious pores can be observed in the cement paste without graphene, and the cement with 0.1% graphene is the most dense. Due to the small amount of graphene incorporated in the experiment, and as the resolution of the electron microscope is 100 nm, it is difficult to observe graphene. However, from the variation in the flexural strength, we can also find that the flexural strength of the cement matrix decreased by 4.87% when the graphene content was 0.01%. The reason may be that the introduction of graphene lead to the poor dispersion effect of graphene and a decline in the flexural strength.

**Figure 4:**
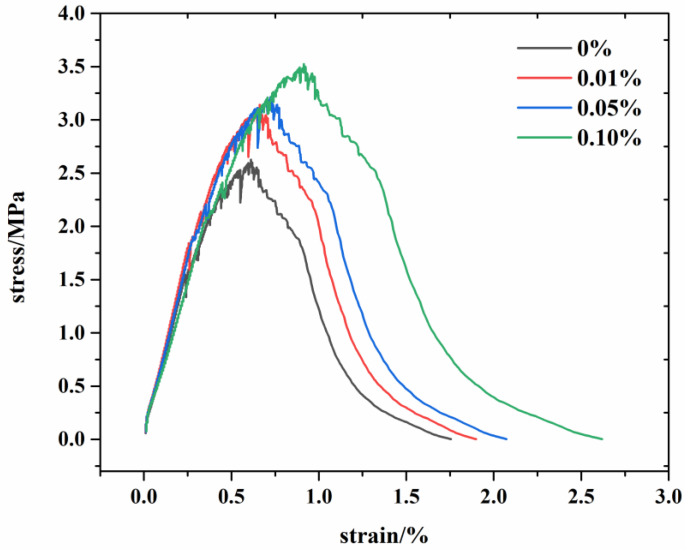
Tensile stress–strain curves of C-S-H and G/C-S-H in the experiment.

At the same time, in order to compare with the results of the molecular simulation, the stress–strain curve of the tensile experiment was obtained (Figure 4). As can be seen from the picture, in the initial stage of the tensile test, that is, when the strain increased from 0 to about 1.0%, the stress rose rapidly until it reached the maximum value, and then the stress began to decrease until it reached 0. And we can find that with the increase in graphene, the strain corresponding to the maximum stress increases gradually, which indicates that the addition of graphene enhances the tensile resistance of the material.

### 3.2. Mechanical Properties of C-S-H in Three Directions

**Figure 5:**
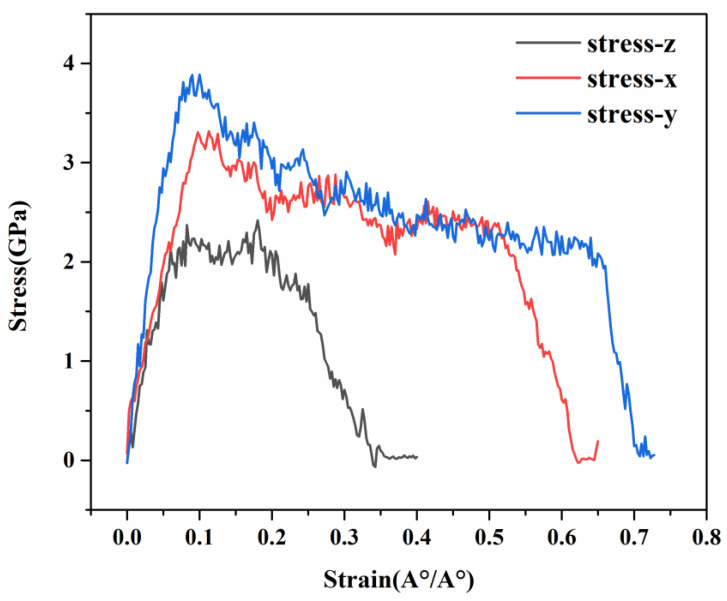
Stress–strain curves of C-S-H in three directions.

Firstly, the tensile properties of C-S-H gel were analyzed. The difference in the uniaxial tensile stress–strain curves in the x, y, and z directions indicates that C-S-H gel has layered structural heterogeneity at the nanoscale. The stress–strain curves show the tensile strength and Young’s modulus in the x, y, and z directions, where the Young’s modulus of C-S-H is equal to the slope of the elastic phase of the stress–strain curve. As shown in Figure 5, in the x direction, the C-S-H structure reaches the tensile strength when the strain is 0.09 Å/Å, the tensile strength is 3.31 GPa, and the Young’s modulus is 36.77 GPa, which is close to the 50 GPa obtained via the nanoindentation test. In the y direction, the C-S-H structure reaches the tensile strength at the strain of 0.07 Å/Å; the tensile strength is 3.74 GPa, and the Young’s modulus is 38.9 GPa, which is close to the 50 GPa obtained by nanoindentation test [29]. In the z direction, the C-S-H structure reaches the tensile strength when the strain is 0.085 Å/Å; the tensile strength is 2.37 GPa, and the Young’s modulus is 27.88 GPa, which is close to the 20–30 GPa obtained from the experiment. At the same time, we can find that the tensile stress in the x and y directions starts to drop sharply at strains of 0.5 Å/Å and 0.65 Å/Å, indicating that the structure begins to fail and then fracture, while the failure in the z direction occurs near the strain of 0.25 Å/Å. The deviations in stress–strain curves in three directions indicate the different mechanisms of heterogeneous deformation of the C-S-H.

It is known that tensile stress refers to the maximum tensile stress that a material can withstand, that is, the maximum stress in the stress–strain curve, while fracture stress refers to the maximum tensile stress that the material can withstand before failure, where the failure of the material is represented by the stage of sharp decline in stress in the stress–strain curve. From Figure 4, we can also note that the fracture stress in the three directions of C-S-H is also different. The fracture stress in the x direction is 2.38 GPa, and the strain is 0.51 Å/Å; the fracture stress in the y direction is 2.06 GPa, and the strain is 0.65 Å/Å; and the fracture stress in the z direction is 1.74 GPa, and the strain is 0.24 Å/Å.

### 3.3. Mechanical Properties of G/C-S-H in Three Directions

**Figure 6:**
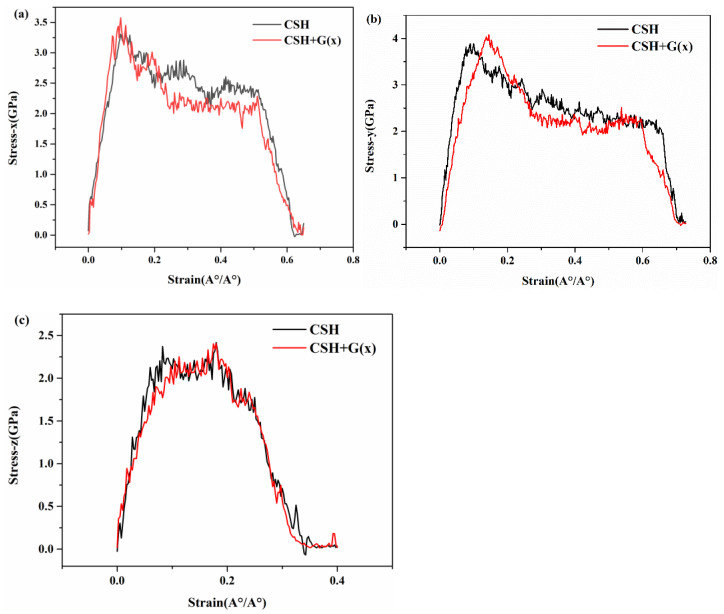
(**a**–**c**) Stress–strain curves in three directions of G/C-S-H (graphene in x direction).

**Figure 7:**
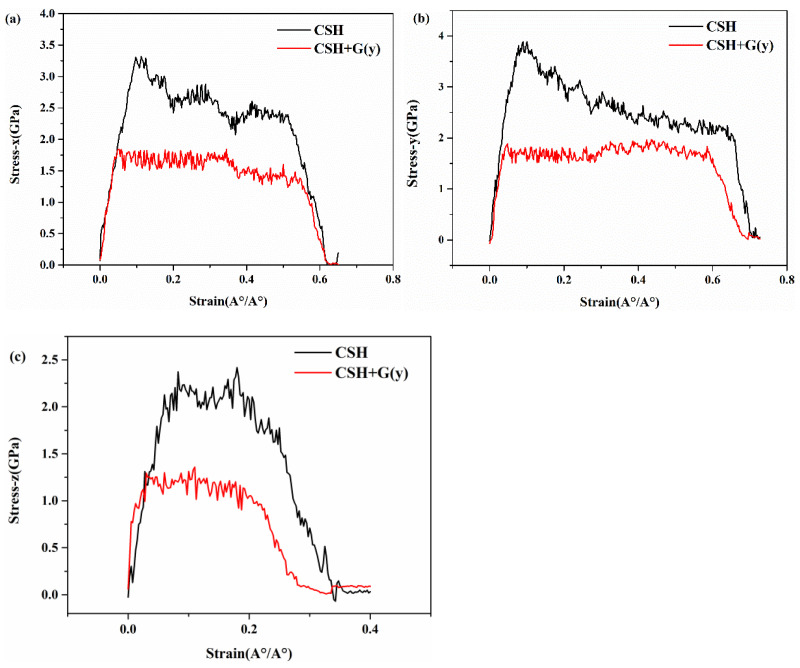
(**a**–**c**) Stress–strain curves in three directions of G/C-S-H (graphene in y direction).

**Figure 8:**
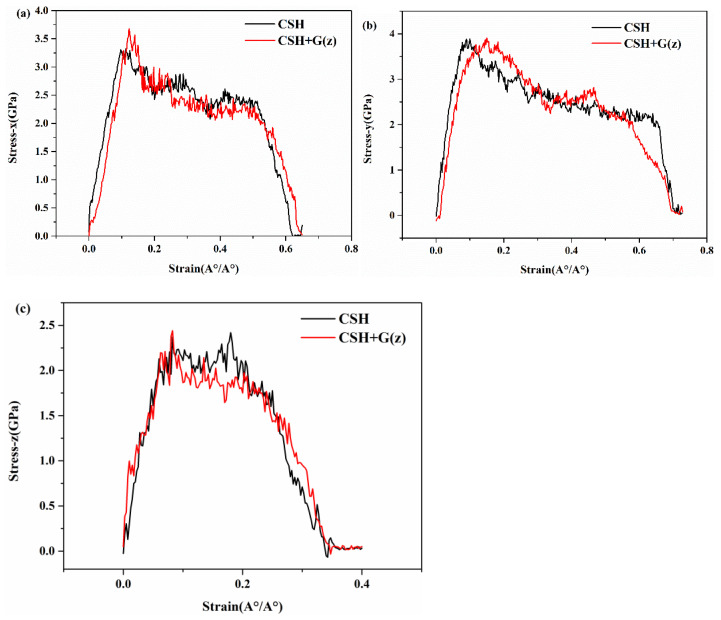
(**a**–**c**) Stress–strain curves in three directions of G/C-S-H (graphene in z direction).

Due to the heterogeneity of the C-S-H gel’s lamellar structure in three directions in the above conclusions, we considered inserting graphene in the x, y, and z directions of the C-S-H structure to study the tensile properties of G/C-S-H. Figure 6, Figure 7 and Figure 8 show the tensile stress–strain curves of G/C-S-H in the x, y, and z directions. First, the graphene structure was inserted in the middle of the x direction of the C-S-H lamellar structure, and the x-direction tensile stress–strain curve of the C-S-H lamellar structure is shown in Figure 6a–c. The trend of stress–strain change was roughly similar to that of C-S-H gel, but the tensile stress of G/C-S-H was improved, and the tensile strength in the x direction is 3.58 GPa. The tensile strength is 8% higher than that in the x direction of the C-S-H. The tensile strength in the y direction is 4.07 GPa, which is 9% higher than that in the y direction of C-S-H. The tensile stress in the z direction is basically the same as that of the C-S-H gel. At the same time, it can be found from Figure 6 that although the addition of graphene in the x direction increases the tensile stress of G/C-S-H, it has almost no effect on the fracture stress.

Secondly, for the structure with graphene inserted in the middle of the y direction of the C-S-H laminate structure, the tensile stress–strain curves in the x, y, and z directions are shown in Figure 7a–c. It can be found from the figures that the variation trend of the stress–strain curves does not change much in the three directions, but the stress peaks in the three directions are significantly reduced. The tensile stress of the G/C-S-H in the x direction is 1.80 GPa, which is 45% less than the tensile stress of C-S-H gel in the x direction. The tensile stress of the G/C-S-H in the y direction is 1.88 GPa, which is 49.7% lower than that of C-S-H gel in the y direction. The tensile stress of the G/C-S-H in the y direction is 1.25 GPa, which is 52.7% lower than that of C-S-H gel in the z direction. From Figure 7, we can also find that the addition of graphene in the y direction not only reduces the tensile stress of G/C-S-H, but also reduces the fracture stress of G/C-S-H.

Finally, for the structure with graphene inserted in the middle of the z direction of the C-S-H laminate structure, the tensile stress–strain curves in the x, y, and z directions are shown in Figure 8a–c. As can be seen from the figures, the tensile stress–strain curves of G/C-S-H are basically consistent with the trend of C-S-H, but the tensile strength of G/C-S-H in the three directions has been improved, and the tensile strength of G/C-S-H in the x direction is 3.67 GPa, which is 11.2% higher than that of C-S-H in the x direction. The tensile strength of G/C-S-H in the y direction is 3.90 GPa, which is 4.3% higher than that of C-S-H in the y direction, and the tensile strength of G/C-S-H in the z direction is 2.44 GPa, which is 3% higher than that of C-S-H in the z direction. It can also be found from Figure 8 that the addition of graphene in the z direction only increased the tensile stress of G/C-S-H, but did not enhance the fracture stress.

By comparing the experimental results (Figure 4) with the molecular simulation results, we can find that they are in good agreement. The tensile strength obtained through the molecular simulation and experiment are about 2.5–4 MPa. The results of molecular simulation show that the insertion of graphene in the x and z directions increases the tensile strength of G/C-S-H, while the insertion of graphene in the y direction decreases the tensile strength of G/C-S-H. However, due to the random insertion of graphene in the experiment, the probability of graphene being present in the x and z directions is greater than that in the y direction, so the tensile strength of G/C-S-H increases.

The insertion of graphene in different directions affects the tensile stress of G/C-S-H for the following reasons: The graphene in the x and z directions is mainly inserted between the interlayer water and interlayer Ca atoms, and the addition of graphene is conducive to the interlayer structure of C-S-H to bond more tightly, but the y direction is the direction of the chain connection of SiO_2_ and Si_2_O_5_, and the insertion of graphene breaks the connection between them, resulting in reductions in the tensile stress and fracture stress of G/C-S-H.

### 3.4. Effect of Temperature and Strain Rate on Mechanical Properties

#### 3.4.1. Effect of Temperature

**Figure 9:**
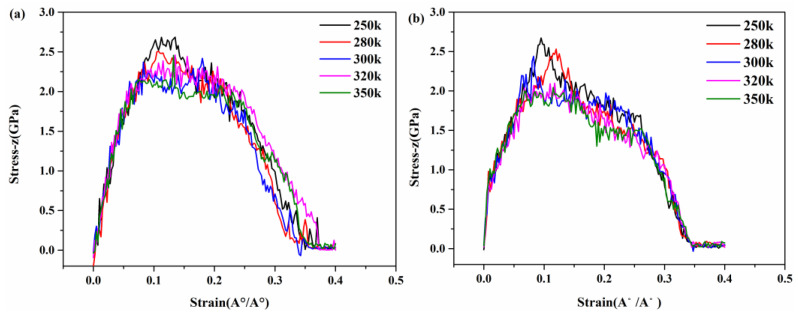
(**a**,**b**) Stress–strain curves of C-S-H and G/C-S-H in z direction at different temperature.

Tensile simulations were performed on the model under different temperatures to study the tensile stress–strain curves. Figure 9a,b give the tensile stress–strain curves in the z direction at the five temperatures of 250 K, 280 K, 300 K, 320 K, and 350 K. In simulations where all other factors are consistent, the temperature difference within 100 K has a limited effect on the tensile stress–strain curve. The effects of C-S-H gel and G/C-S-H at different temperatures are compared. Overall, temperature has a similar effect on C-S-H and G/C-S-H. The tensile strength decreased slowly with the increase in temperature. As can be seen from the figures, the tensile strength of C-S-H decreases from 2.50 GPa to 2.13 GPa when the temperature increases from 250 K to 350 K, and the tensile strength of G/C-S-H decreases from 2.67 GPa to 2.18 GPa when the temperature increases from 250 K to 350 K. The stress–strain curve in Figure 9 also shows that the fracture stress of G/C-S-H gradually decreases with the increase in temperature.

The reason for the above changes is that at a higher temperature, the thermal movement of the material atoms is more intense, and the atoms of the material are constantly vibrating and colliding, which leads to changes in the internal structure of the material, and the interaction force between the atoms is also changed, resulting in a reduction in the strength of the material.

#### 3.4.2. Effect of Loading Rate

**Figure 10:**
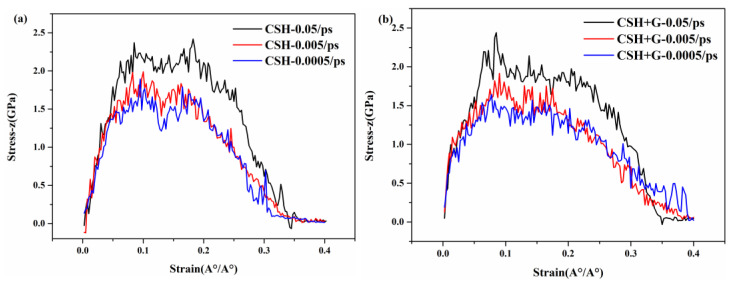
(**a**,**b**) Stress–strain curves of C-S-H and G/C-S-H in z direction at different loading rates.

As shown in Figure 10a,b, the z-direction tensile simulation of C-S-H and G/C-S-H was run to study the effect of loading rate on mechanical properties. It can be found that the effect of loading rate on stress–strain curves of C-S-H and G/C-S-H show the same tendency. The tensile strength increases with the increase of the loading rate. This is because atomic diffusion and crack evolution are limited at higher loading rates. This is also consistent with the research conclusions of macroscopic experiments: Rossi et al. found through experiments that the cement materials showed better tensile strength under a high loading rate [21]. At the same time, due to the high loading rate, the water in the micropores of the C-S-H and G/C-S-H produces reflux force on the solid, thus increasing the failure strength, which is also one of the reasons that the high loading rate improves the tensile properties. The stress–strain curve in Figure 10 also shows that with the increase in loading rate, the fracture stress of G/C-S-H also increases.

### 3.5. Effect of Defect of Graphene on Mechanical Properties

**Figure 11:**
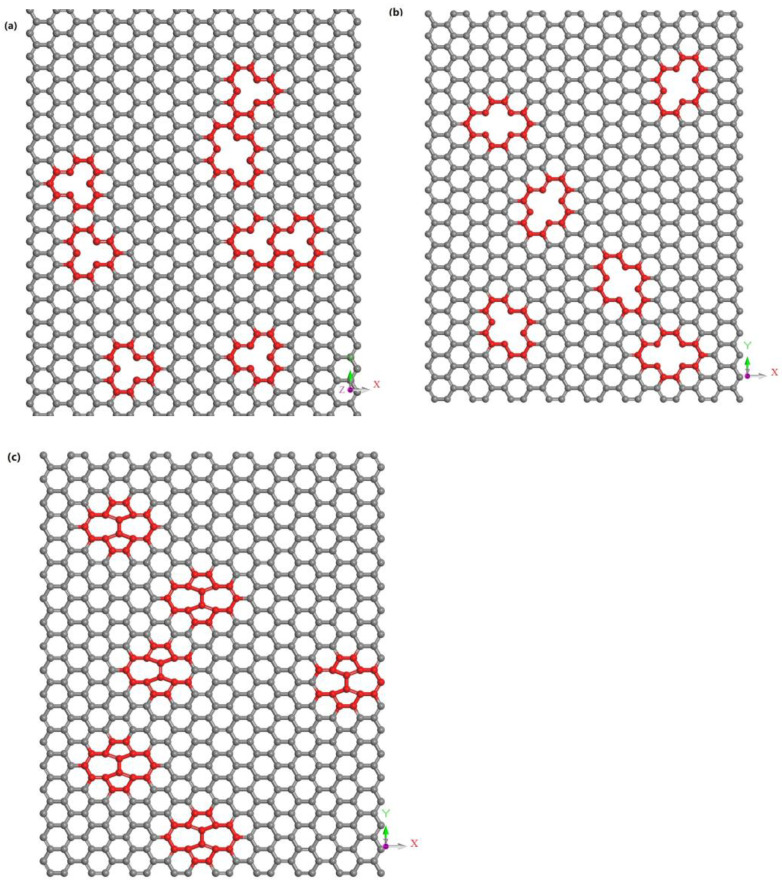
(**a**–**c**) The monovacancy defects, divacancy defects, and S-W defects with random orientation without stress.

**Figure 12:**
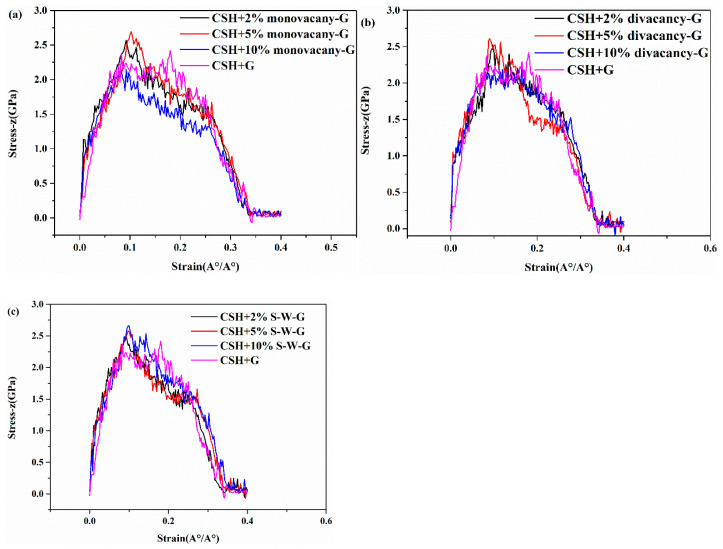
(**a**–**c**) Stress–strain curves of G/C-S-H in z direction with different graphene defects.

Then, we introduced three types of defects on the graphene: the monovacancy defect, divacancy defect, and S-W defect are shown in Figure 10, Figure 11 and Figure 12. The monovacancy defect and divacancy defect are constructed by introducing a loss of one carbon atom and two adjacent carbon atoms into the model, as shown in Figure 11a,b. The S-W defect, shown in Figure 11c, is formed by two adjacent carbon atoms being rotated 90° around their central point, so that the original four hexagonal rings are rotated into two pentagonal rings and two heptagonal rings. Therefore, it can also be called the 5-7-7-5 defect. Each defective graphene has the following defect concentration conditions: 2%, 5%, and 10%. The defect concentration value here is obtained by dividing the number of non-hexagonal rings in the defect-containing graphene by the number of hexagons in the perfect graphene [30]. The distribution of the location and direction of the defects is random. Finally, the statistical average is used as the final result to make the results convincing. Figure 12a–c show the tensile stress–strain curves of G/C-S-H samples with three types of defects with three different concentrations.

As for monovacancy defects, it can be seen that defects have a certain influence on tensile strength. The introduction of monovacancy defects increases the tensile strength of G/C-S-H slightly, but with the increase in defect concentration, the tensile strength shows a small decrease. With the increase in strain, the effect of defects on the tensile properties of G/C-S-H can be ignored. The same tendency can be found for the effect of the divacancy defect on the tensile properties of G/C-S-H. It can be seen from the figure that the tensile strength of monovacancy vacancy defects and diatomic vacancy defects at different concentrations is ordered as follows: $f_{5\%}>f_{2\%}>f>f_{10\%}$. In the case of the monovacancy defect, the maximum tensile strength is 2.69 GPa and the maximum increase is 10.2%. In the case of the divacancy defect, the maximum tensile strength is 2.60 GPa and the maximum increase is 6.6%. For S-W defects, the tensile stress of G/C-S-H increases with the increase in defect concentration. It can be seen from the figure that the tensile stress of S-W defects at different concentrations is ordered as follows: $f_{10\%}>f_{5\%}>f_{2\%}>f$. The maximum tensile strength is 2.66 GPa with an increase of 9%.

The reason for the above phenomenon may be that monovacancy defects and divacancy defects of 2% and 5% concentrations can make the binding of C-S-H with graphene closer, thus transferring the load of C-S-H to the graphene with better tensile properties through the interface between graphene and C-S-H. However, defects of 10% concentration will not only weaken the binding of C-S-H with graphene, but also reduce the mechanical properties of graphene itself, thereby reducing the mechanical properties of G/C-S-H. For S-W defects, there is no absence of carbon atoms, so the mechanical properties of graphene itself do not decrease with the increase in defect concentration. In addition, the change of hexagonal rings into pentagonal and heptagonal rings in graphene also changes its energy field, which may allow C-S-H to bind more tightly to graphene, and therefore enhance the mechanical properties of G/C-S-H.

## 4. Conclusions

The mechanical properties and intrinsic interactions of G/C-S-H composites have been studied at both micro and macro levels using a combination of macroscopic experiments and MD simulations, from which the following conclusions can be drawn:

1. The experimental results show that in the G/C-S-H composites, the compressive, flexural, and tensile strengths were increased by up to 21.9%, 12.19%, and 34.6%, respectively. This was attributed to the bridging effect of graphene on the control of microcracks in the cement matrix.
2. The uniaxial tensile mechanical properties of C-S-H and G/C-S-H in the three directions were simulated using molecular dynamics methods, and the results show that the C-S-H gel exhibited different mechanical properties in the three directions. The tensile strength of C-S-H in the x, y, and z directions is 3.31 Gpa, 3.74 Gpa, and 2.37 Gpa, respectively. The uniaxial tensile stress–strain curve of C-S-H in the x direction is basically consistent with the trend in the y direction, and the tensile stress in the z direction is lower, which is caused by the different layered structures of the C-S-H in the three directions.
3. By inserting graphene in the x, y, and z directions of C-S-H, the tensile process of G/C-S-H was simulated. The results showed that the insertion of graphene in the x and z directions increased the tensile strength in the three directions, while the insertion of graphene in the y direction decreased the tensile strength of the G/C-S-H in the three directions.
4. The results of uniaxial tensile simulations of C-S-H and G/C-S-H at different temperatures show that the tensile stress decreases slowly with increasing temperature. Meanwhile, the uniaxial tensile strength of C-S-H and G/C-S-H increases with the increase in loading rate.
5. The effects of three kinds of defects and concentrations of graphene on the uniaxial tensile properties of G/C-S-H were studied. The tensile stress of monovacancy vacancy defects and diatomic vacancy defects at different concentrations is ordered as follows: $f_{5\%}>f_{2\%}>f>f_{10\%}$. And the tensile strength of S-W defects at different concentrations is ordered as follows: $f_{10\%}>f_{5\%}>f_{2\%}>f$.

## Data Availability

Data are contained within the article.
